# Spectral Processing for Denoising and Compression of 3D Meshes Using Dynamic Orthogonal Iterations

**DOI:** 10.3390/jimaging6060055

**Published:** 2020-06-26

**Authors:** Gerasimos Arvanitis, Aris S. Lalos, Konstantinos Moustakas

**Affiliations:** 1 Department of Electrical and Computer Engineering, University of Patras, 26504 Patras, Greece; arvanitis@ece.upatras.gr (G.A.); moustakas@ece.upatras.gr (K.M.); 2 Industrial Systems Institute, ATHENA Research and Innovation Center, 26504 Platani-Patras, Greece

**Keywords:** spectral processing, dynamic orthogonal iterations, compression and denoising of 3D meshes

## Abstract

Recently, spectral methods have been extensively used in the processing of 3D meshes. They usually take advantage of some unique properties that the eigenvalues and the eigenvectors of the decomposed Laplacian matrix have. However, despite their superior behavior and performance, they suffer from computational complexity, especially while the number of vertices of the model increases. In this work, we suggest the use of a fast and efficient spectral processing approach applied to dense static and dynamic 3D meshes, which can be ideally suited for real-time denoising and compression applications. To increase the computational efficiency of the method, we exploit potential spectral coherence between adjacent parts of a mesh and then we apply an orthogonal iteration approach for the tracking of the graph Laplacian eigenspaces. Additionally, we present a dynamic version that automatically identifies the optimal subspace size that satisfies a given reconstruction quality threshold. In this way, we overcome the problem of the perceptual distortions, due to the fixed number of subspace sizes that is used for all the separated parts individually. Extensive simulations carried out using different 3D models in different use cases (i.e., compression and denoising), showed that the proposed approach is very fast, especially in comparison with the SVD based spectral processing approaches, while at the same time the quality of the reconstructed models is of similar or even better reconstruction quality. The experimental analysis also showed that the proposed approach could also be used by other denoising methods as a preprocessing step, in order to optimize the reconstruction quality of their results and decrease their computational complexity since they need fewer iterations to converge.

## 1. Introduction

Nowadays, due to the easiness of creating digital 3D content, a great amount of information can be easily captured and stored instantly. However, the information acquired by 3D scanners is usually huge and unorganized, creating noisy and dense 3D models that are very difficult to be efficiently handled by other high-level applications and software (e.g., 3D object recognition [1,2], 3D matching and retrieval [3], scalable coding of static and dynamic 3D objects [4], re-meshing [5], etc.) without further processing (i.e., compression and denoising). This increasing interest for 3D meshes has affected many different scientific areas and industries, such as mobile cloud gaming and entertainment [6], heritage culture [7], medicine [8], 3D tele-immersion, communication [9,10] and more.

Spectral methods have been excessively used in the image, video, and signal processing domains trying to solve low-level problems by manipulating the eigenvalues, eigenvectors, eigenspace projections, derived from the graph Laplacian operator. In the same way, spectral methods can be utilized for the processing of 3D meshes consisting of connected vertices. However, the computational complexity and the memory requirements of these methods strongly depend on the density of the 3D model, resulting in to become prohibitive when the number of vertices significantly increases. As it has been suggested in [11,12], this issue can be addressed if the raw geometry data were divided and processed separately in blocks representing different overlapping parts of a mesh, namely submeshes.

More specifically, the direct implementation of the Singular Value Decomposition (SVD) method on the graph Laplacian of each submesh, has an extremely high computational complexity, requiring $O\left(n^{3}\right)$ operations, where *n* denotes the number of vertices in a 3D mesh. Motivated by this drawback, we propose an approach that is based on a numerical analysis method known as orthogonal iterations (OI) [13], that takes advantage of the geometric coherence between different submeshes of the same mesh. The method starts by separating the 3D mesh into different submeshes and then it uses the corresponding spectral values of a previous submesh to readjust only a small number of spectral coefficients of a next submesh. In this way, we achieve a significant speed-up since it requires $O\left(nc^{2}\right)$ where *c* is the number of the preserving spectral components c<<n. Additionally, we developed a dynamic OI approach that automatically estimates the ideal value of *c* so that to achieve a specifically wanted reconstruction quality based on predefined thresholds.

The rest of this paper is organized as follows. Section 2 presents previous works related to spectral processing methods in 3D meshes. Section 3 introduces some basic definitions and presents in detail the proposed orthogonal iteration and approach. In Section 4, we discuss the dynamic approach that automatically identifies the optimal subspace size of *c*, satisfying predefined reconstruction quality constraints. In Section 5, we investigate the spatial coherence between submeshes of the same mesh. We also study the impact of the submesh size on the reconstruction quality and the computational complexity of the proposed approach. Section 6 presents the use cases in which the proposed method utilized (i.e., compression and denoising in static and dynamic 3D meshes). In Section 7, we evaluate the performance of the proposed method, using different 3D models, and finally, Section 8 draws conclusions about the method.

## 2. Previous Works

Several surveys that cover basic definitions and applications of the graph spectral methods have been introduced by Gotsman [14], Levy [15], Sorkine [16], Vallet and Levy [17] and more recently by Zhang et al. [5]. All these surveys classify the spectral methods according to several criteria related to the employed operators, the application domains and the dimensionality of the spectral embeddings used.

Graph Spectral Processing (GSP) of 3D meshes is based on the singular/eigenvectors and/or eigenspace projections derived from appropriately defined mesh operators. There is a big variety of different tasks in which GSP has been used, such as implicit mesh fairing [18], geometry compression [16,19] and mesh watermarking [20]. Taubin [21] was the first that treated the coordinate vertices of a 3D mesh as a 3D signal, introducing the graph Laplacian operators for discrete geometry processing. The similarities between the spectral analysis concerning the mesh Laplacian and the classical Fourier analysis motivated him for this analysis. A summary of the mesh filtering approaches that can be efficiently carried out in the spatial domain using convolution approaches is given [22].

Despite their applicability in a wide range of applications such as denoising, compression and watermarking, they require the computation of explicit eigenvector making them prohibitive for real-time scenarios. Additionally, there are a lot of applications in literature in which large-scale 3D models are scanned in parts [23,24,25] providing in this way a consecutive sequence of coherent 3D surfaces that need to be processed fast. Our method has been designed in order to be ideally suited particularly in these cases, providing accurate results while the whole process takes part in real-time.

Computing the truncated singular value decomposition can be extremely memory-demanding and time-consuming. To overcome these limitations, subspace tracking algorithms have been proposed as fast alternatives relying on the execution of iterative schemes for evaluating the desired eigenvectors per incoming block of floating-point data corresponding in our case, to different surface patches [26]. The most widely adopted subspace tracking method is the Orthogonal Iterations (OI) since it provides very fast solutions when the initial subspace, which is given as input, is close enough to the subspace of interest. Additionally, the size of the subspace remains at a small level [27]. The fact that both matrix multiplications and QR factorizations have been highly optimized for maximum efficiency on modern serial and parallel architectures, makes the OI approach more attractive for real-time applications.

This work is an extended version of the research presented in [28]. In this version, we provide more details about the ideal mesh segmentation (e.g., number of submeshes, size of overlapped submehses) and the submeshes properties (e.g., spatial coherence between submeshes of the same mesh). Additionally, we extend the application scenarios presenting a block-based spectral denoising approach for 3D dynamic meshes.

## 3. Spectral Processing Using Orthogonal Iterations

### 3.1. Preliminaries of Spectral Processing in 3D Meshes

In this work, we assume the use of a triangle mesh M with *n* vertices $v_{i}=\left(x_{i},\;y_{i},\;z_{i}\right)\;\forall \;i=1,\cdots ,n$ and $n_{f}$ faces $f_{i}=\left\{v_{i1},\;v_{i2},\;v_{i3}\right\}\;\forall \;i=1,\cdots ,n_{f}$, represented by its corresponding centroids $m_{i}=\left(v_{i1}+v_{i2}+v_{i3}\right)/3\;\forall \;i=1,\cdots ,n_{f}$. In this way, the mesh can be represented by two different sets $M=\left(V,F\right)$ corresponding to the vertices *V* and the indexed faces *F* of the mesh. Spectral processing approaches, applied in 3D meshes [16,19], usually decompose the Laplacian matrix L trying to take advantage of the special characteristics that the eigenvalues and eigenvectors can provide. The Laplacian matrix L can be calculated according to:

(1) $$\begin{array}{ccc} L & = & D-A \end{array}$$

where $A\in R^{n\times n}$ can represent a binary or a weighted adjacency matrix like the following:

(2) $$A_{ij}=\left\{\begin{array}{cc} \frac{1}{∥v_{i}-v_{j}{∥}_{2}^{2}} & \left(i,j\right)\in E\\ 0 & otherwise \end{array}\right.$$

where *E* is a set of edges that can be directly derived from *V* and *F* and the matrix D is the diagonal matrix where the non-zero elements are estimated as $D_{ii}=\sum_{j=1}^{n}A_{ij}\;\forall \;i=1,\cdots ,n$.

In contrast to the binary adjacency matrix, which provides only connectivity information, the weighted adjacency matrices are ideal for emphasizing the geometrical and topological coherence between the connected vertices. The decomposition of the matrix L can be estimated as:

(3) $$L=U\Lambda U^{T}$$

where $\Lambda =\left\{\lambda_{1},\lambda_{2},\ldots ,\lambda_{n}\right\}$ is a diagonal matrix consisting of the eigenvalues of L that can be considered as graph frequencies, and $U=[u_{1},\ldots ,u_{n}]$ is the matrix with the eigenvectors $u_{i}\in R^{n\times 1}$ [16] that demonstrate increasing oscillatory behavior as the magnitude of $\lambda_{i}$ increases [29]. The Graph Fourier Transform (GFT) of the vertices is defined as the projection of the corresponding coordinates onto the matrix of the eigenvectors according to:

(4) $$\bar{v}=U^{T}v$$

Correspondingly, and the inverse GFT (IGFT) can be estimated according to:

(5) $$v=U\bar{v}$$

### 3.2. Block-Based Spectral Processing Using Orthogonal Iterations

The decomposition of the graph Laplacian, using a direct SVD implementation, is prohibitive for very dense meshes. To overcome this drawback, several approaches have been presented in the literature. Many of these approaches propose to separate the 3D meshes into smaller parts [12,30] and then to handle each one of these parts separately. Following this line of thought, we suggest the partitioning of the original large mesh into *k* parts using the MeTiS algorithm described in [31]. To be able to directly apply OI, we require to process sequentially a series of matrices of the same size. To that end, we create overlapped equal-sized submeshes of $n_{d}$ vertices, as described in Section 5.1 and Section 5.4. In this case, the process for the decomposition of the $L\left[i\right]\;\forall \;i=1,\ldots ,k$ requires $O(k{n_{d}}^{3})$ floating-point operations, which is also computational high and not acceptable for use in real-case scenarios. To overcome this problem, minimizing the computational complexity, we suggest using the processing output of a submesh as input for the orthogonal iteration process of a next submesh taking advantage of the coherence between the spectral components of the different submeshes [32], since the initialization of OI to a starting subspace close to the subspace of interest leads to a very fast solution. The assumption, concerning the coherence, is based on the observation that submeshes of the same mesh maintain similar geometric characteristics and connectivity information, which will be further discussed in Section 5.2.

The Orthogonal Iteration is an iterative procedure that computes the singular vectors corresponding to the dominant singular values of a symmetric, non-negative definite matrix [33]. To make the process more computational light, we suggest to preserve the *c* eigenvectors corresponding to the *c* lowest eigenvalue of $U_{c}\left[i\right]=\left[u_{1},\ldots ,u_{c}\right]\in R^{n_{d}\times c}$ for each *i* submesh, according to Algorithm 1:
**Algorithm 1:** Orthogonal Iteration updating process for the *i*th submesh
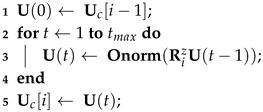

where $R_{i}={\left(L\left[i\right]+\delta I\right)}^{-1}$ and δ denotes a very small positive scalar value ensuring the positive definiteness of the matrix $R_{i}$ and matrix $I\;R^{n_{d}\times n_{d}}$ denotes the identity matrix. The equation:

(6) $$R_{i}^{z}U\left(t-1\right)$$

is estimated very efficiently using sparse linear system solvers, as described in [16]. The value of the power *z* plays an important role to the converge of the process that will be analyzed in following section. The convergence rate of OI depends on $|\lambda_{c+1}/\;\lambda_{c}{|}^{z}$ where $\lambda_{c+1}$ is the (c+1)-st largest eigenvalue of $R_{i}$ [13]. The initial subspace $U_{c}\left[0\right]$ has to be orthonormal in order to preserve orthonormality. For this reason, $U_{c}\left[0\right]$ is estimated by a direct SVD implementation, while all the following subspaces $U_{c}\left[i\right]$, i=2,…,k are estimated by adjustation, as presented in the Algorithm 1.

Several widely adopted methods, such as the Householder Reflections (HR), Gram-Schmidt (GS) and Modified Gram Schmidt (MGS) [34], perform orthonormalization of the estimated subspace. In this work the Onorm(·) step is performed as follows:

(7) $$\begin{array}{cc} R^{z}\left[i\right]U_{c}\left[i\right] & \Rightarrow Q_{qr}\left[i\right]R_{qr}\left[i\right]\\ U_{c}\left[i\right] & =Q_{qr}\left[i\right]=\left[Q_{qr}{\left[i\right]}_{\left(:,1\right)},\ldots ,Q_{qr}{\left[i\right]}_{\left(:,c\right)}\right] \end{array}$$

where matrix $Q_{qr}\left[i\right]$ is evaluated by applying *c* sequential HR reflections. Therefore, $Q_{qr}\left[i\right]$ is the submatrix that corresponds to the first *c* columns of:

(8) $$\begin{array}{c} Q_{qr}\left[i\right]=H_{1}^{T}\cdot H_{2}^{T}\cdot \ldots \cdot H_{c}^{T} \end{array}$$

## 4. Dynamic Orthogonal Iterations for Stable Reconstruction Accuracy

In use cases where the ground truth model is known beforehand (i.e., compression), we can use this knowledge to provide a dynamic pipeline that automatically identifies the optimal subspace size *c* (i.e., ideally number of remaining low-frequency components) that satisfies a specific quality requirement. This dynamic process takes into account a predefined threshold that determines the preferable perception quality of the reconstructed mesh. When we provide an initialization that is closer to the real solution, then the final results have more perceptual quality and in this way, the error between the reconstructed and the ground truth object is reduced. The method is based on the observation that the feature vectors $E\left[i\right]=U_{c}^{T}\left[i\right]v\left[i\right]$ of each submesh v[i] has different subspace $U_{c}\left[i\right]$ size and it should be carefully selected so that to have the minimum loss of information.

We estimate the following mean residual vector e(t), in order to quantify the loss of information in each *t* iteration:

(9) $$e\left(t\right)=\sum_{j\in \left\{x,y,z\right\}}\left(v_{j}\left[i\right]-U_{c}\left[i\right]U_{c}^{T}\left[i\right]v_{j}\left[i\right]\right)$$

Then, we assume that when the $l_{2}$-norm of the metric e(t) is lower than a given threshold ${∥e\left(t\right)∥}_{2}<\epsilon_{h}$ then the perceptual loss is decreased and in this case the reconstructed result is assumed as acceptable. To reduce the residual error e(t), we suggest adding one normalized column in the estimated subspace $U_{c}\left(t\right)=U_{c}\left(t-1\right)\;\;e\left(t-1\right)/{∥e\left(t-1\right)∥}_{2}$ and then perform orthonormalization is estimated according to:

(10) $$U_{c}\left(t\right)=\mathrm{Onorm}\left\{R^{z}\left[i\right]\left[U_{c}\left(t-1\right)\;\;\frac{e(t-1)}{{∥e\left(t-1\right)∥}_{2}}\right]\right\}$$

On the other hand, if the $l_{2}$-norm of the metric e(t) is less than a pre-defined threshold $\epsilon_{l}$ then subspace size is decreased by 1 by simply selecting the first $c_{i}-1$ columns of $U_{c}\left(t\right)$. This is an iterative procedure that automatically stops when the metric ${∥e\left(t\right)∥}_{2}$ lies between the range of the thresholds $\left(\epsilon_{l},\epsilon_{h}\right)$, where the threshold $\epsilon_{l}$ represents the lowest and $\epsilon_{h}$ represents the highest allowed value. This means that if the value of ${∥e\left(t\right)∥}_{2}$ is lower or higher of the aforementioned range then we need to increase or decrease it, respectively, according to the rules that are clearly presented in the Algorithm 2. To mention here that this process gives the flexibility to a user to easily trade his/her preference between the reconstruction quality and the computational complexity, just changing the values of the preferable thresholds. The following Algorithm 2 summarizes the steps of the proposed approach.
**Algorithm 2:** Dynamic Orthogonal Iterations applied in the *i*th submesh
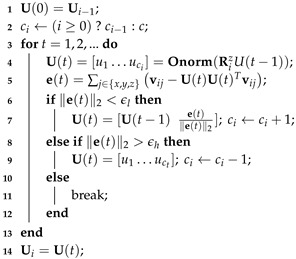


## 5. Ideal Mesh Segmentation and Submeshes Properties

In this section, we study the impact of the submesh size to the reconstruction quality and the execution time. Additionally, we present the methodology that we follow for the final reconstruction of the mesh meaning that the submeshes are overlapped and some points appear in more that one submesh. The section is concluded with some experimental results confirming the validity of the assumption about the spatial coherence between submeshes of the same meshes.

### 5.1. Weighted Average for Mesh Reconstruction and Guarantees of a Smooth Transition

**Figure 1:**
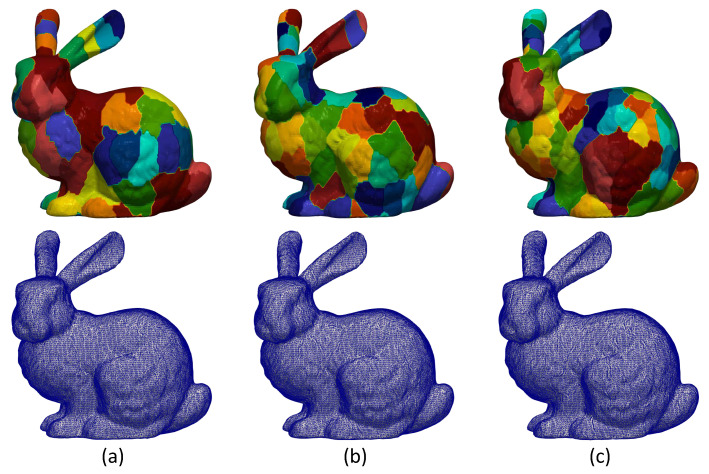
(**First line**) Segmentation of bunny model using MeTis algorithm in (**a**) 70, (**b**) 100 and (**c**) 200 parts. (**Second line**) The corresponding reconstructed models without applying overlapping process (edge effect is apparent).

As we have mentioned earlier, a mesh is separated into different submeshes and then they are processed seperately, using spectral techniques. However, in this case, the final reconstructed model has a loss of quality that is attributed to the dislocation of the vertices lying in the areas where two submeshes have common edges. This phenomenon is known as edge effect (see Figure 1) and it requires special treatment in order to be mitigated or eliminated. To overcome this problem, we create overlapped submeshes [12,30,35] extending each submesh using also neighboring vertices of the boundary nodes of adjacent submeshes until fulfilling a predefined number of $n_{d}$ vertices, in total, for all submeshes of the mesh. This operation reduces the error introduced and additionally creates equal-sized submeshes which are necessary for the proceeding of the OI. In Figure 1, we present different segmentation scenarios using MeTis algorithm. Inspecting the second line of this figure, which presents the reconstructed model highlighting the edges of the triangles, it is apparent that the more the parts of the segmentation are, the more apparent the edge effect is.

**Figure 2:**
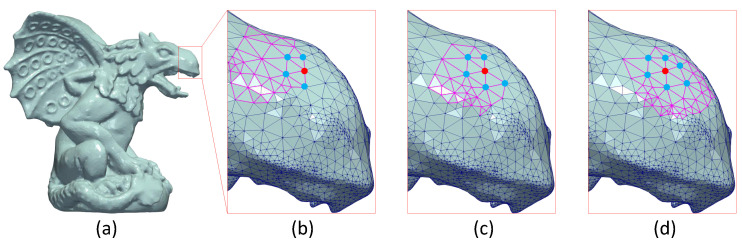
The red point has different degree in each submesh of the (**a**) original model (Gargoyle model), the corresponding weights are: (**b**) w = 4, (**c**) w = 5, (**d**) w = 6.

The edge effect is attributed to missing neighbors inevitably caused by the mesh segmentation. Missing neighbors means missing connectivity which resulting in missing entries in the graph Laplacian matrix. However, an efficient way to deal effectively with this limitation is to combine the reconstructed geometry of the overlapped parts. The weights that are assigned to each point are proportional to the degree of the node (e.g., number of neighbors) in the corresponding submesh. Overlapping ensures that each vertex will participate in more than one submesh, and thus the probability of having the same degree (in at least one of them) significantly increases. In Figure 2, we present an example showing the weights assigned to a point (highlighted in red) that participates in three overlapped submeshes. The steps that are followed for the estimation of the weighted average coordinates of the overlapped points, are presented in Algorithm 3.
**Algorithm 3:** Weighted average process for the reconstruction of a mesh
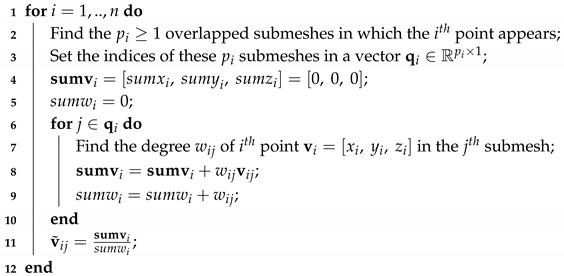


Additionally, we investigate whether the segmentation and the processing of the overlapped patches guarantee the smooth transition in different cases where edge points belong to flat or sharp areas. At this point it should be mentioned that, the edge points could be part of edges, corners or flat areas. In the following, we present results showing that the way we treat the edge points guarantees, in all the aforementioned cases, a smooth transition successfully mitigating the edge effects.

**Figure 3:**
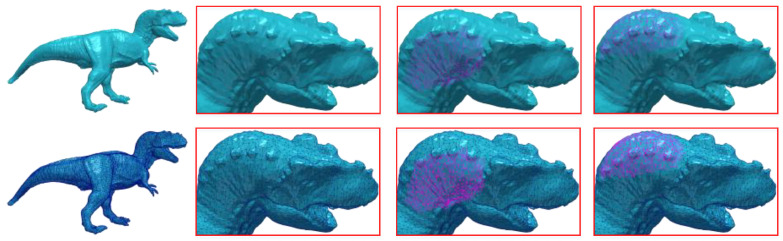
Overlapped parts means that each boundary point belongs to more than one part and its degree may vary significantly between different parts.

The process starts using the MeTis algorithm for the identification of the initial parts. Then each part is extended, using the neighbors of the boundary nodes that belong to adjacent parts until all of them has the same predefined size. Consequently, each boundary point participates in more than one segments. The weights that are assigned to each point, which participates in more than one parts, represents its degree (i.e, the number of connected neighbors) in the specific part (see Figure 3). The final position of an edge point is evaluated using the weighted average approach as mentioned above.

We show the distribution of error in the internal and the boundary points of each submesh. For this specific study we consider three different cases that are described below:

- Non Overlapping case, where each node participates in only one part.
- Overlapping case, where each part is extended using the neighbors of the boundary nodes that belong to adjacent parts. Thus, each boundary point participates in more than one parts, which are reconstructed individually. The final position of a boundary point is evaluated using the simple average of the reconstructed positions.
- Weighted Overlapping case, where each part is extended using the neighbors of the boundary nodes that belong to adjacent parts and the final position of a boundary point is evaluated using a weighted average. The weights assigned to each point that participates in more than one parts, represent its degree (i.e, the number of its neighbors) in the specific part.

**Figure 4:**
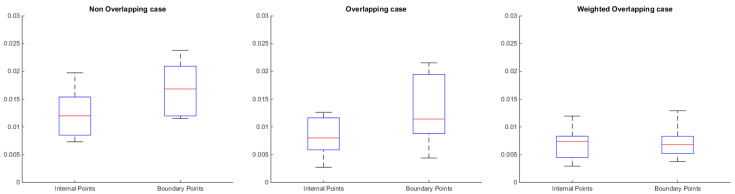
Standard deviation of the reconstructed error in the internal and the boundary points of each submesh for each one of the aforementioned cases.

The standard deviation of the reconstructed error in the internal and the boundary points of each submesh for each one of the aforementioned cases is provided in Figure 4. For the creation of this figure, we used eight models in total (fandisk, armadillo, block, tyra, twelve, bunny, gargoyle, Julio) and we took into account the reconstructed error per each patch of all models. On each box, the central mark is the median, the edges of the box are the 25th and 75th percentiles, and the whiskers extend to the most extreme data points that are not considered outliers. By inspecting this figure, it can be clearly shown that the weighting scheme guarantees a smooth transition, since the distribution of error in the internal and boundary points has almost identical characteristics, significantly outperforming the other two cases.

**Figure 5:**
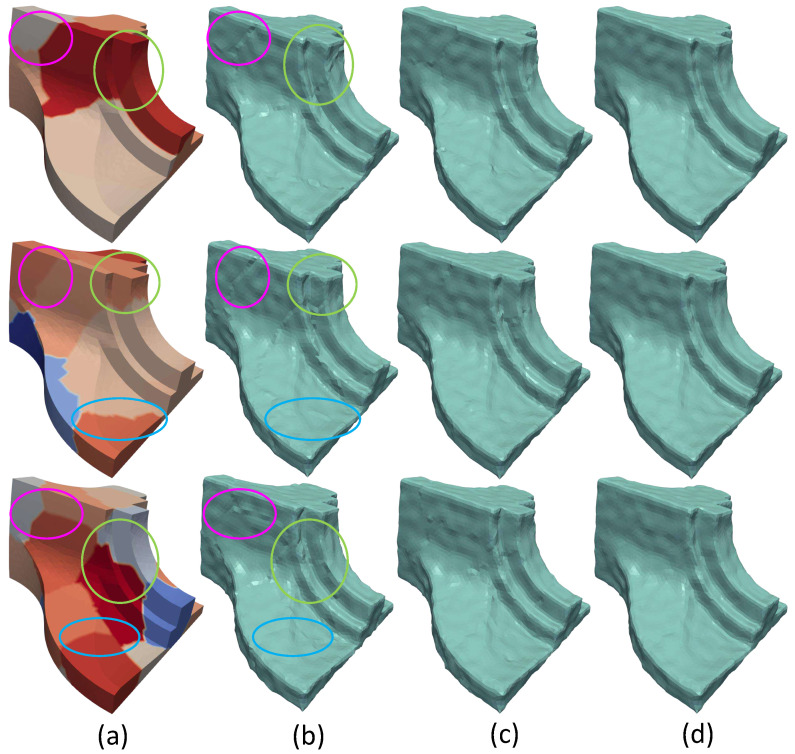
(**a**) The model separated in different number of parts (10, 15 and 20, respectively). Additionally, indicative areas have been selected where two or more submeshes are connected; (**b**) Non Overlapping case, the edge effect is apparent in areas where submeshes are connected; (**c**) Overlapping case, the edge effect have been mitigated but have not been eliminated yet. The bigger the number of the partitioning the more intense the problem of the effect; (**d**) Weighted Overlapping case, the results seem to be independent and unaffected of the partitioning (Fandisk $\sigma^{2}=0.2$).

Similar conclusions can be also perceived by observing the Figure 5. In this figure, the results of a coarse denoising step are presented after partitioning Fandisk model in a different number of submeshes (10, 15 and 20, respectively). It is obvious that the error on the boundary nodes is minimized in the weighted average case, while the segmentation effects are very noticeable in the other two cases.

### 5.2. Spatial Coherence between Submeshes of the Same Mesh

The previously presented approach, using OI for the estimation of matrices $U_{c}\left[i\right]\;\forall \;i=1,\cdots ,k$, strongly depends on the assumption that there is a spatial coherence between submeshes of the same mesh. Supposing the correctness of this assumption, the matrix $U_{c}\left[i-1\right]$, which is used for initializing Algorithm 1, is the best-related approximation meaning that its form is very close to the real solution. The best-provided initialization matrix has as a result a faster convergence, providing at the same time the most reliable results. In this approach, the proposed initialization strategy suggests using as initial estimation the solution of the previous adjacent submesh. At the following, we will study the validity of this assumption via extensive simulations using different models. Our study is based on the observation that the surface’s form of a mesh follows the same pattern, which means that neighboring parts of the same mesh have:

(i) Similar connectivity properties (degree and distance).
(ii) Same geometrical characteristics which are shared between connected points (curvature, small-scale features, texture pattern etc.).

**Figure 6:**
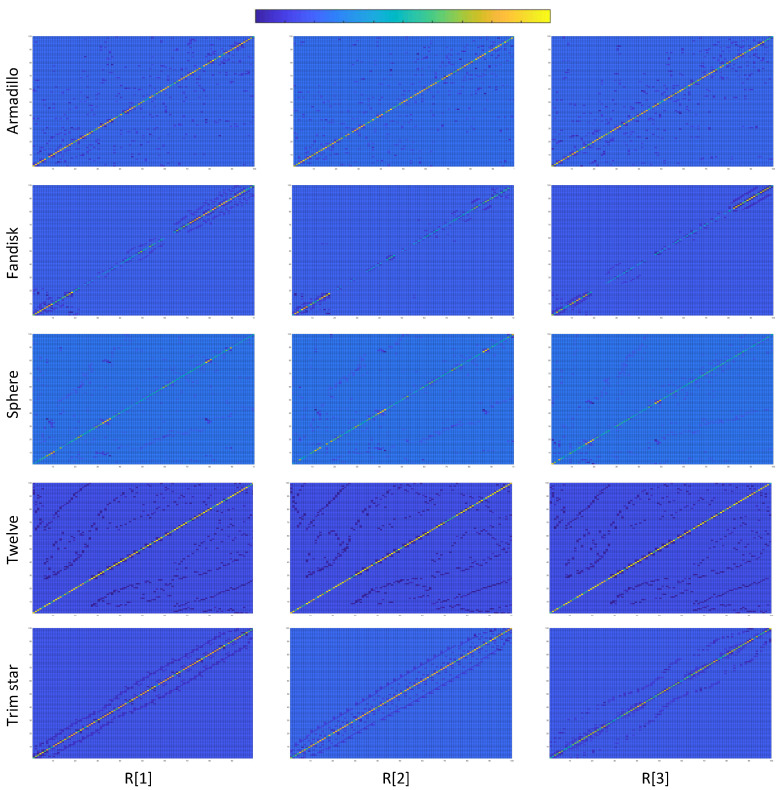
Laplacian matrices of different submeshes for different models in color based on the values of their cells. It can be easily observed that different submeshes of the same model follow a similar form while they are totally different in comparison with submeshes of different meshes.

Figure 6 presents colored images representing the Laplacian matrices R[i]∀i={1,2,3} of different submeshes for several 3D models. Providing an easier comparison between the images, we have created matrices of submeshes with the same size 100×100 so that $R\in R^{100\times 100}$. Each pixel (x,y) of an image represents the corresponding color coded value of R(x,y). Additionally, a color bar is also provided showing the range of colors between the lowest and the highest value of each matrix R, where, the deep blue represents the lowest value of each matrix while the bright yellow represents the highest value. We can observe that different submeshes of the same model follow a similar form while they are totally different in comparison with submeshes of different meshes.

Similar conclusions could be perceived by observing the Table 1. Each row of this table presents the Mean Squared Error (MSE) estimated by the comparison between the random matrix R of a model, represented as R[1], and the mean matrix $\tilde{R}$ of any other model which appear in Figure 6, including the mean matrix of the same model. This comparison is repeated using different models (other rows of this table). For the sake of simplicity, we used only one random matrix R[1]. However, similar results are extracted using any other random matrix of a model.

### 5.3. Number of Submeshes

The ideal selected number of submeshes depends on the total number of points of the mesh. Large submeshes create large matrices increasing significantly the processing time since the number of edge points increases. On the other hand, using small submeshes the final results are negatively affected by the edge effects. Table 2 shows how the number of segments affects the metric of Mean Normal Difference (MND) for both averaging cases (simple and weighted average), where MND represents the average distance from the resulting mesh normals to the ground truth mesh surface.

**Figure 7:**
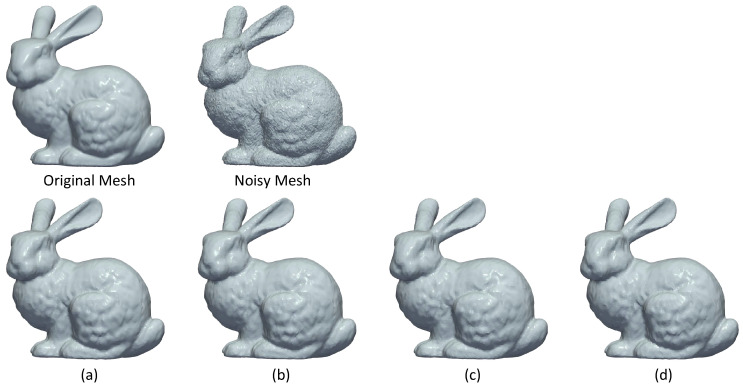
(**First line**) Original and Noisy mesh. (**Second line**) Coarse denoising meshes separated by Metis in (**a**) 25 submeshes, (**b**) 50 submeshes, (**c**) 70 submeshes, (**d**) 100 submeshes.

In Figure 7, the results of coarse smoothing, using a different number of segments, are also presented. As we can observe, there is no remarkable visual difference between the reconstructed models. Additionally, if we consider the fact that these results could be further improved by the use of a fine denoising step then the number of segments is not a critical factor.

### 5.4. Size of Overlapped Submeshes

The real motivation behind the processing in parts, is strongly supported by the existence of a great amount of state-of-the-art applications in which large 3D models cannot be scanned at once using portable 3D scanners. As a result, the output of the sequential scanning would be a sequence of submeshes that arrive sequentially in time. An extensive evaluation study carried out using different overlapped sizes (Table 3, Table 4 and Table 5) showed that the reconstruction quality is strongly affected by the size of the submeshes themselves rather than the number of overlapped vertices.

**Figure 8:**
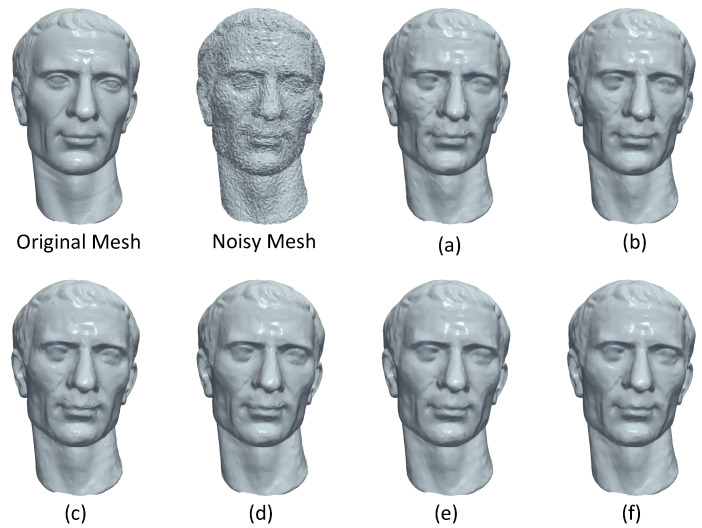
Coarse denoising meshes with 70 equal-sized overlapped submeshes consisting of (**a**) 532 vertices (max), (**b**) 558 vertices (1.05 · max), (**c**) 585 vertices (1.10 · max), (**d**) 611 vertices (1.15 · max), (**e**) 638 vertices (1.20 · max), (**f**) 665 vertices (1.25 · max).

Regarding the ideal size of the overlapped patches, we investigated the effect of using different sizes of overlapped submeshes in a range from 5 to 25% of the maximum submeshes length, in the quality of the reconstructed model. More specifically, as shown in Table 4 and Table 5 and in Figure 8, the mean normal difference and the visual smoothed results have not significant differences between the different case studies, especially for percentages up to 10% of the max segment. Additionally, if we consider the fact that this process takes place in the coarse denoising step we can conceive the negligible contribution of the overlapped submeshes size to the final denoising results.

By inspecting the results, we can definitely state that the number and size of segments are much more important than the size of the overlapped patches. The overlapping process mainly contributes in the case of on-the-edge points helping for a more accurate estimation of their position by creating full-connected points. A sufficient overlapping size corresponds to the 15% of the total points in the submesh.

Figure 8 illustrates the reconstruction results of the coarse denoising step using 70 overlapped submeshes consisting of a different number of vertices in each case. As we can observe, in cases where the number of overlapping vertices is higher than 15% of the total number of submesh points then the reconstructed results are almost identical with the 15% case.

## 6. Case Studies

In this section, we will present how the proposed approach could be used in different applications, such as compression [36] and denoising [37], speeding up the computational efficiency of their spectral processing part (both for static and dynamic meshes).

### 6.1. Block-Based Spectral Compression

In the literature, a lot of works have been presented related to compression of 3D meshes and point clouds [38,39,40]. The spectral compression methods utilize the subspace of the eigenvector matrix $U_{c}\left[i\right]$ for encoding the geometry of a 3D mesh. This matrix can be computed by a direct SVD implementation or by executing a number of orthogonal iterations on $R^{z}\left[i\right]$, and it is used as the encoding dictionary to provide a compact representation of the vertices of each submesh.

- At the encoder: The coordinates $v_{x}\left[i\right]\in R^{n_{d}\times 1}$ are projected to the dictionary and we finally take the feature vector E according to:
  (11) $$E\left[i\right]=U_{c}^{T}\left[i\right]v\left[i\right]$$
  where $E\left[i\right]\in R^{c\times 1}$ and $c<<n_{d_{i}}$.
- At the decoder: The inverse process takes place, the vertices of the original 3D mesh are reconstructed from the feature vector $E\left[i\right]$ and the dictionary $U_{c}\left[i\right]$ according to:
  (12) $$\tilde{v}\left[i\right]=U_{c}\left[i\right]E\left[i\right]$$

The sender transmits only the connectivity of the mesh and the *c* respective spectral coefficients of each block. On the other hand, the receiver evaluates the dictionary $U_{c}\left[i\right]$, based on the received connectivity, and uses the spectral coefficients to retrieve the coordinates of the original mesh $\hat{x},\hat{y},\hat{z}$ [19]. To mention here that the subspace size *c* has a fixed value in the case of OI, providing fast execution times but having a lack of reconstruction accuracy. On the other hand, the DOI approach provides reconstructed results with high and stable reconstruction quality, since it searches for the “ideal” subspace size, but as a result, it adds an extra computational cost.

### 6.2. Block-Based Spectral Denoising

**Figure 9:**
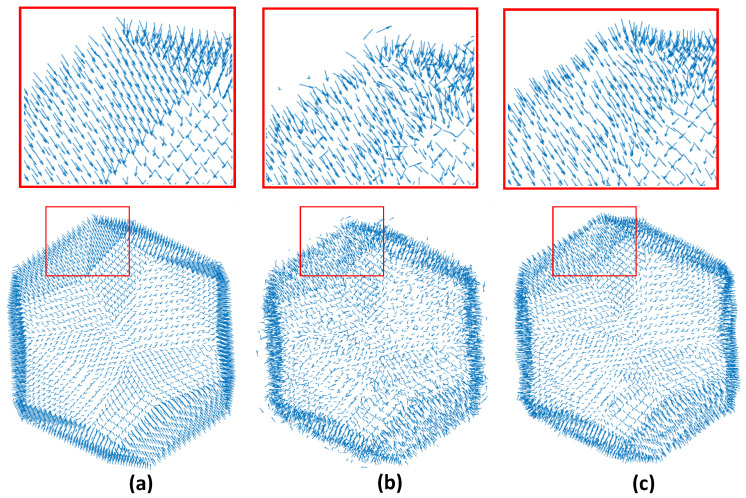
Face normals of: (**a**) The original noise-free mesh; (**b**) the noisy mesh; (**c**) Smoothed reconstructed model.

The proposed spectral denoising approach is separated into two stages (i.e., coarse and fine). Firstly, the coarse step filters out the high spectral frequencies, and then a bilateral approach [41,42,43] performs fine denoising. The coarse step is used to accelerate the convergence of the fine step since it mitigates the noise that appears in the high-frequency components. This provides a set of face normals that are closer to the face normals of the original model, as shown in Figure 9.

The fine denoising step starts having as input the vertices $\hat{v}\left[i\right]=U_{c}U_{c}^{T}v\left[i\right]$ of the coarse denoised *i* submesh, the centroid $m_{i}$ of each face, and the corresponding face normals that are estimated according to:

(13) $$\begin{array}{c} {\hat{n}}_{m_{i}}=\frac{\left({\hat{v}}_{i_{2}}-{\hat{v}}_{i_{1}}\right)\times \left({\hat{v}}_{i_{3}}-{\hat{v}}_{i_{1}}\right)}{∥\begin{array}{c} \left({\hat{v}}_{i_{2}}-{\hat{v}}_{i_{1}}\right)\times \left({\hat{v}}_{i_{3}}-{\hat{v}}_{i_{1}}\right) \end{array}∥}\;\forall \;i=1,n_{f} \end{array}$$

where ${\hat{v}}_{i_{1}}$, ${\hat{v}}_{i_{2}}$, ${\hat{v}}_{i_{3}}$ represents the connected vertices that constitute the face $f_{i}$ and ${\hat{n}}_{m}=\left[{\hat{n}}_{m_{1}}^{T}\;{\hat{n}}_{m_{2}}^{T}\cdots \;{\hat{n}}_{nf}^{T}\right]\in R^{3n_{f}\times 1}$. The main purpose of the bilateral filtering is to estimate the new noise-free face normals ${\hat{n}}_{m_{i}}$, according to:

(14) $${\hat{n}}_{m_{i}}=\frac{1}{W_{i}}\sum_{f_{j}\in N_{f_{i}}}A_{j}K_{s}\left(m_{i},m_{j}\right)K_{r}\left(n_{m_{i}},n_{m_{j}}\right)n_{m_{j}}$$

where $N_{f_{i}}$ is the set of faces that have a common edge with the face $f_{i}$, $A_{j}$ is the area of face $f_{j}$, $W_{i}$ is a weight for ensuring that the vector ${\hat{n}}_{m_{i}}$ is a unit vector and $K_{s}$, $K_{r}$ are some Gaussian kernels, as presented in the next equations:

(15) $$\begin{array}{ccc} K_{s}\left(m_{i},m_{j}\right) & = & exp\left(-\frac{{∥m_{i}-m_{j}∥}^{2}}{2\sigma_{s}^{2}}\right) \end{array}$$

(16) $$\begin{array}{ccc} K_{r}\left(n_{m_{i}},n_{m_{j}}\right) & = & exp\left(-\frac{{∥n_{m_{i}}-n_{m_{j}}∥}^{2}}{2\sigma_{r}^{2}}\right) \end{array}$$

Then, the fine-denoised face normal ${\hat{n}}_{m_{i}}$ is used to update the vertex positions in order to match to the new normal directions $n_{m_{i}}$ in an iterative manner, according to:

(17) $$\begin{array}{ccc} {\hat{v}}_{i_{j}}^{\left(t+1\right)} & = & {\hat{v}}_{i_{j}}^{\left(t\right)}+\frac{1}{\left|F_{i_{j}}\right|}\sum_{z\in F_{i_{j}}}{\hat{n}}_{m_{z}}\left[{\hat{n}}_{m_{z}}^{T}\left(m_{i}^{\left(t\right)}-{\hat{v}}_{i_{j}}^{\left(t\right)}\right)\right] \end{array}$$

(18) $$\begin{array}{ccc} m_{i}^{\left(t\right)} & = & \left({\hat{v}}_{i_{1}}^{\left(t\right)}+{\hat{v}}_{i_{2}}^{\left(t\right)}+{\hat{v}}_{i_{3}}^{\left(t\right)}\right)/3 \end{array}$$

where (t) represent the number of the iteration and $F_{i_{j}}$ denotes the vertices of the first-ring area of the vertex ${\hat{v}}_{i_{j}}$.

#### Bilateral Filter as a Graph-Based Transform

In this subsection, we will show how the fine denoising step of the aforementioned approach (i.e., bilateral filtering) can be also considered as a graph spectral processing approach. We start assuming the existence of an undirected graph G=(V,E) where the nodes $V=\left\{1,2,\ldots ,n\right\}$ are the normals $n_{m_{i}}$, associated with the centroids $m_{i}$ and the edges *E* capture the similarity between two normals as given by the bilateral weights in Equations (15) and (16). The input normals can be considered as a signal defined on this graph $n_{i}:V\to R^{3\times 1}$ where the signal value at each node correspond to the normal vector. Considering the weighted adjacency matrix C, consisting of the bilateral weights, and the diagonal matrix $D=diag\left\{W_{1},\ldots ,W_{n_{f}}\right\}$, then the Equation (14) can be written as:

(19) $$\begin{array}{ccc} \hat{n} & = & D^{-1}Cn\\ \; & = & D^{-1/2}D^{-1/2}CD^{-1/2}D^{1/2}n\\ D^{1/2}\hat{n} & = & \left(I-L\right)D^{1/2}n\\ D^{1/2}\hat{n} & = & \underset{IGFT}{\underbrace{U}}\underset{\begin{array}{c} Spectral\\ response \end{array}}{\underbrace{\left(I-\Lambda \right)}}\underset{GFT}{\underbrace{U^{T}}}D^{1/2}n \end{array}$$

Equation (19) confirms our assumptions that the Bilateral filter can be considered as a frequency selective graph transform with a spectral response that corresponds to a linear decaying function, meaning that it tries to preserve the low-frequency components and attenuate the high-frequency ones.

### 6.3. Block-Based Spectral Denoising of 3D Dynamic Mesh

In previous sections, we mentioned that the Laplacian matrices of submeshes, representing parts of the same 3D model, have similar form confirming the existence of spatial coherence. As we presented, we can take advantage of this property implementing a more efficient OI process providing both faster convergence and more accurate results.

However, the advantages of this approach could be better highlighted in the dynamic case. A dynamic mesh consists of s frames/meshes which are shared the same connectivity with each other. Apparently, the Laplacian matrices of corresponding submeshes R[i]∀i=1,…,k are preserved the same, without changing, by frame to frame (e.g., $R_{1}\left[1\right]=R_{2}\left[1\right]=\cdots =R_{s}\left[1\right])$, where $R_{j}\left[i\right]$ represents the Laplacian matrix of the *i*th submesh of the *j*th frame.

**Figure 10:**
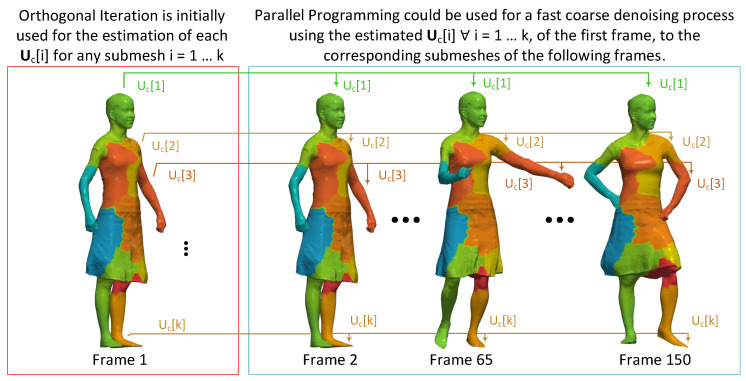
Parallel programming schema for high-performance coarse denoising of a 3D dynamic mesh.

Figure 10 illustrates a schema representing the proposed coarse denoising of a dynamic mesh. The process starts by iteratively applying OI for the estimation of each $U_{c}\left[i\right]\;\forall \;i=1,\ldots ,k$, as detailed described in Algorithm 1. Then, parallel programming could be used for a fast coarse denoising process taking advantage of the already estimated matrices. In this case, the denoising process can run for all frames concurrently because no information of the previous frames is required (except of the matrices $U_{c}\left[i\right]\;\forall \;i=1,\ldots ,k$ which are estimated once during the OI process applied only to the first frame). Additionally, adaptive compression of animated meshes could be used for real-time scenarios, as described in [44].

### 6.4. Comparisons of the Execution Times with a Relative Method

In this subsection, we present the execution time effectiveness of our method in comparison with the relevant method of Vallet and Levy [17].

The main contribution of Vallet and Levy’s method is the development of an efficient numerical mechanism that computes the eigenfunctions of the Laplacian. The eigenfunctions are computed band by band based on spectral transforms and an efficient eigensolver, and also using an out-of-core implementation that can compute thousands of eigenvectors for meshes with up to a million vertices. They also propose a limited-memory filtering algorithm, that does not need to store the eigenvectors. Vallet and Levy’s method is very fast, especially in comparison with the traditional SVD decomposition and it also shares a lot of common ideas with our method, trying to solve a similar problem. The main similarity between Vallet and Levy’s method and our approach is that both of them can be used as low-pass filtering.

Nevertheless, Vallet and Levy’s method has some limitations that our method can efficiently handle and overcome. More specifically:

- Their method is not able to preserve the creases and as a future extension, they suggested the use of eigenfunctions of an anisotropic version of the Laplace operator that could improve the frequency localization of the creases and therefore to better preserve them when filtering. We overcome this limitation by using an extra stage of processing (called as a fine step) that handles each area with an anisotropic way taking into account the different geometrical characteristics of small surfaces (e.g., creases, corners, edges, etc.)
- Another limitation is the fact that Vallet and Levy’s method cannot be directly applied to mesh compression since they took particular care, making their Laplacian geometry dependent. On the other hand, our method can be efficiently used for mesh compression as also many experimental results can verify.
- Regarding the performance of the computational complexity, Vallet and Levy’s forecast that partitioning also partially fixes the problem of spatial localization at the expense of losing continuity (this is also why JPEG uses small blocks instead of applying the DCT to the whole image). Their suggestion can be verified by our implementation since we achieve tremendously faster execution times by participating in patches the whole 3D mesh and proceed them separately.

**Figure 11:**
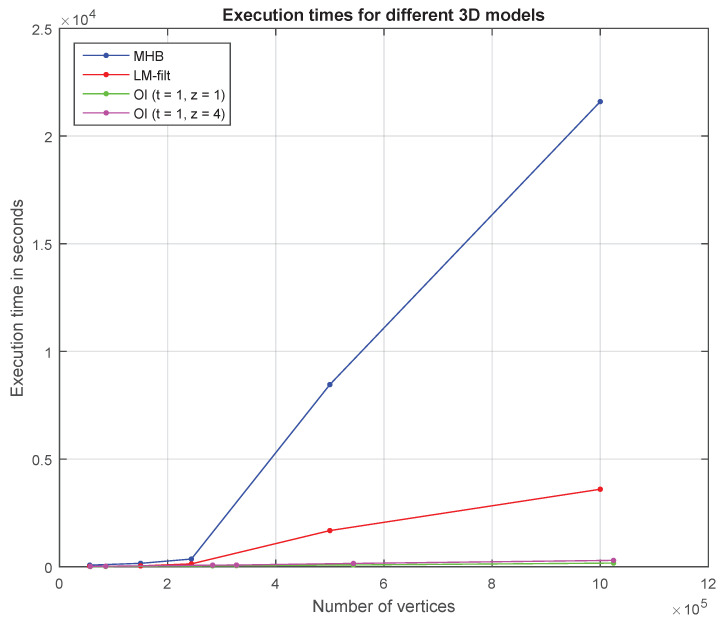
Execution times for different 3D models.

Figure 11 depicts plots that show the execution times of two OI approaches (i.e., OI (*t* = 1, *z* = 1) and OI (*t* = 1, *z* = 4)) in comparison with the execution times of the Manifold Harmonics basis (MHB) and Limited-memory MH Filtering (LM-filt), as presented in [17]. The main reason why our method is much faster than other decomposition methods is due to the fact that it handles many but much smaller matrices (of submeshes) than the large Laplacian matrix of the initial whole mesh. The execution time to decompose a matrix exponentially increases as the dimension of the matrix also increases. On the other hand, the cumulative time to decompose many but small matrices is significantly lower.

## 7. Experimental Results and Evaluation

In this section, we evaluate the results and the performance of the proposed approach in two different use cases, namely compression and denoising.

### 7.1. Experimental Setup and Metrics

The quality of the reconstructed models is evaluated using (i) the normalized mean square visual error (NMSVE) [19] that captures the distortion between the original and the reconstructed model and (ii) the metric θ that represents the mean angle difference between the face normals of the the original and the reconstructed model. The assumption that the noisy 3D object has the same connectivity with the original is only used for the evaluation of the reconstructed mesh with the ground truth. Anywise, our method is not negatively affected by the form or the accuracy of the connectivity, but only cares about how the noisy vertices are connected with their neighbor vertices.

### 7.2. Experimental Analysis of the Spectral Compression Approach

**Figure 12:**
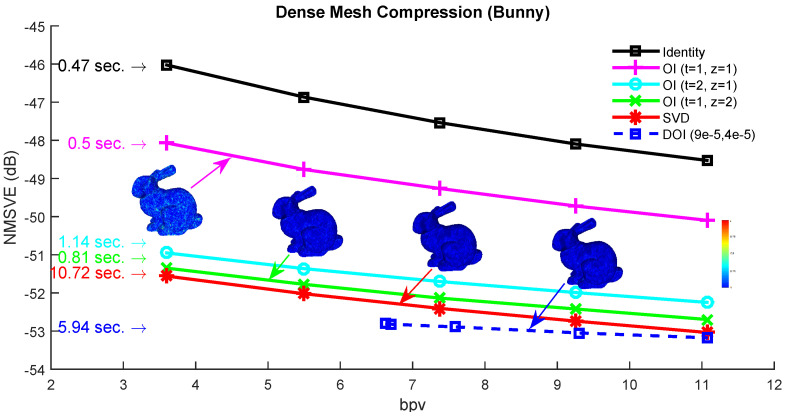
Normalized mean square visual error (NMSVE) of the reconstructed models per different bpv for different compared approaches.

The spectral compression approach is performed as described in Section 6.1. Figure 12 shows how the selected rate of bit per vertex (bpv) affects the metric NMSVE for different compared approaches. We also provide the execution times, next to each line, that encapsulates the respective time needed to run each method (e.g., to construct the matrix $R^{z},z\geq 1$ and execute the OI).

As we can also conclude by observing this figure, OI performs almost the same reconstructed quality with the SVD method, in considerably less time, since it can be executed up to 20 times faster. It is obvious that the more the number of the iterations of OI, the better the reconstructed accuracy of the model, converging towards the (optimal) SVD result. Obviously, this strategy increases the total execution time due to the more iterations, however, the total execution of OI still remains much faster than this one of the direct SVD.

**Figure 13:**
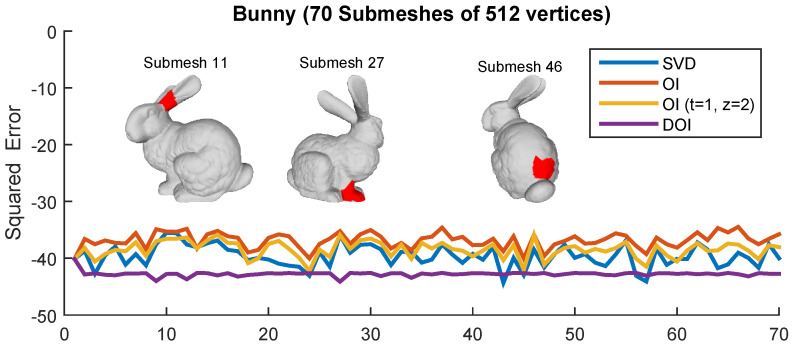
Squared error per each submesh for different approaches.

**Figure 14:**
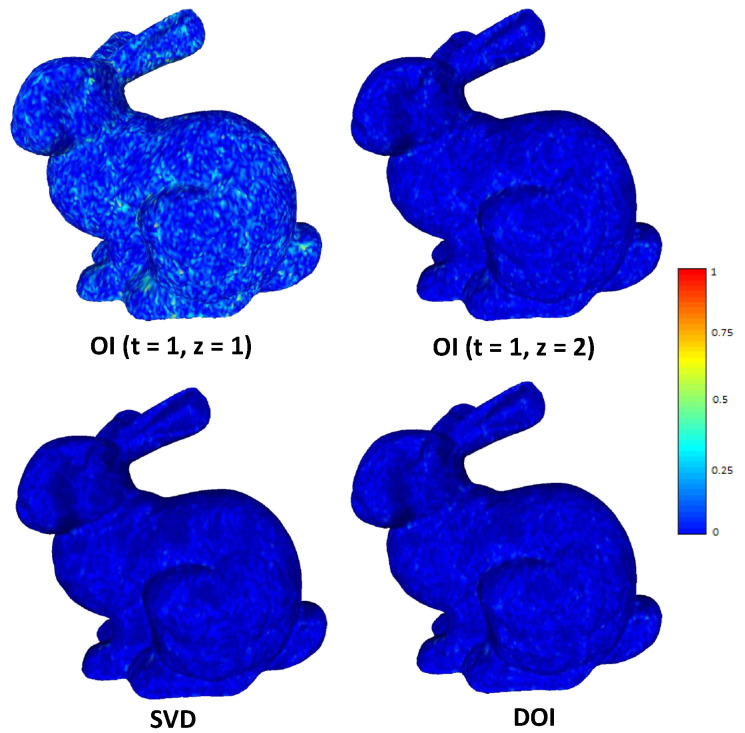
Heatmap visualazation of normal difference with bpv 5.64 for different reconstructed approaches.

For the case of DOI, there is an increase in the execution time, in comparison to OI. However, it still is significantly faster than the SVD (it needs almost half time). On the other hand, there is a significant increase in the final compression rate (bpv), which is captured as a right shifting of the plot in Figure 12. The shifting is more obvious when the initial value of *c* is small which means that more iterations are necessary for achieving the satisfying accuracy. The theoretical complexity of the proposed schemes is in tandem with the measured time. More specifically, the OI approach for the “Bunny” model can be executed much faster than the direct SVD approach. While running more OI iterations yields a better NMSVE, converging towards the (optimal) SVD result, it comes at the cost of a linear increase in the decoding time. On the other hand, one iteration of $R^{4}$ achieves the same visual error as executing four OI, in considerably less time. Figure 13 plots the squared error per each submesh of the Bunny model (70 submeshes in total). Each presented approach has a different reconstruction performance in different submeshes except of the DOI that provides a stable reconstruction accuracy due to the “ideal” value of subspace size that is required to satisfy a predefined reconstruction quality threshold. Figure 14 presents the heatmap visualization of the normals’ difference between the ground truth and the reconstructed models for different OI approaches and SVD.

**Figure 15:**
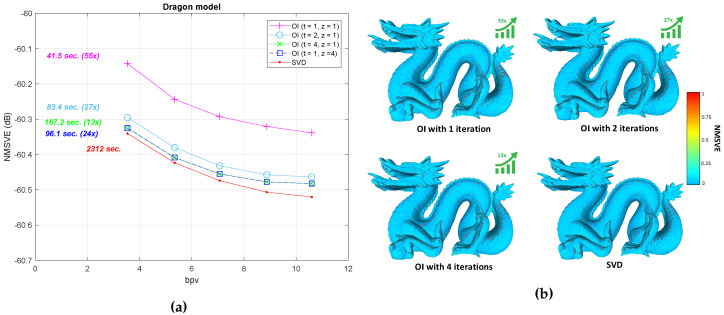
(**a**) NMSVE vs bpv plot of the Dragon model (437,645 vertices); (**b**) normal difference with bpv 7.06. The arrow represents the speed-up in execution time compared to SVD.

The plot of bpv vs. NMSVE for the “Dragon” model is shown in Figure 15a. Note that the execution times shown next to each line encapsulate the respective time needed to construct $R^{z},z\geq 1$, and to run the respective number of OI, with the speed-up as compared to SVD shown in parenthesis. By inspecting the figure, it can be easily concluded that the quality of the OI method performs almost the same as with SVD, especially when the number of iterations increases. Additionally, in Figure 15b, we provide the heatmap visualization of the normals difference between the ground truth and the reconstructed models for different OI approaches and SVD.

### 7.3. Experimental Analysis of Spectral Denoising Approach

**Figure 16:**
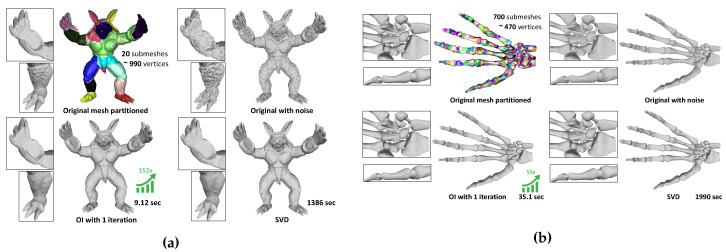
Filtering of 3D models (**a**) Armadillo (20,002 vertices) using c=297 and (**b**) Hand (327,323 vertices) using c=47 with noise: N(0,0.2). Each color represents a submesh, and the arrow depicts the speed-up in execution time compared to SVD.

For the spectral denoising approach, we followed the same steps as these that are described in Section 6.2. OI was used as a pre-processing “smoothing” step before applying the fine spectral bilateral filtering. In Figure 16, we present the smoothed results (“Armadillo” and “Hand” models) between the OI approach and SVD. As we can see, the results of the two methods seem identical. The direct correlation between the size of a block and the execution time speed-up as compared to direct SVD is also highlighted here. For this scenario, zero-mean Gaussian noise N(0,0.2) was added to the models. The “Armadillo” model was partitioned into 20 submeshes each comprising around 990 vertices, while the “Hand” model was partitioned into 700 submeshes with 470 vertices per block.

**Figure 17:**
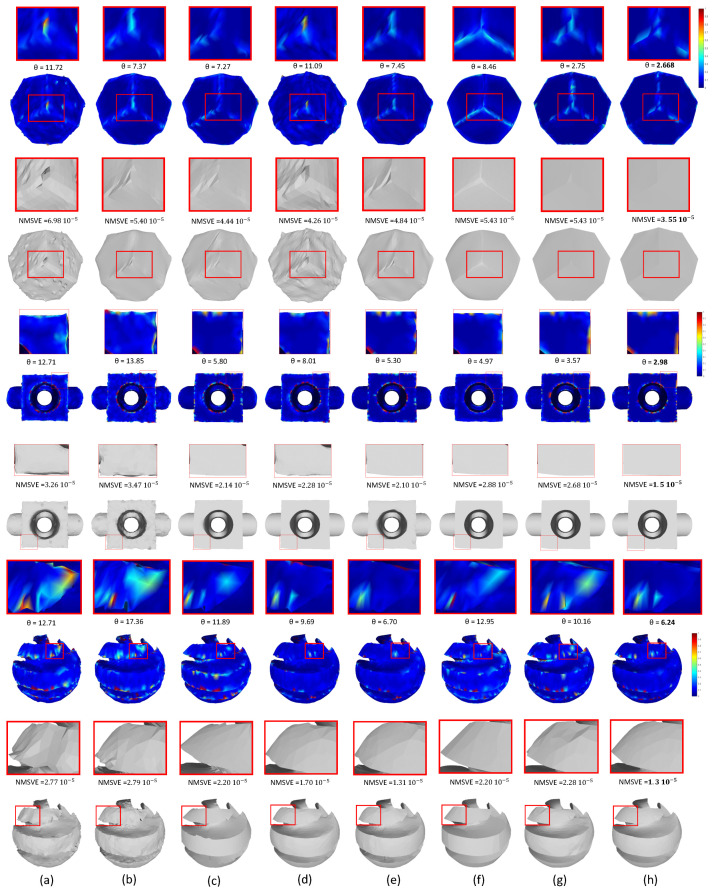
Denoising results for different methods and heatmap visualization; (**a**) bilateral [41]; (**b**) non-iterative [45]; (**c**) Fast and Effective [46]; (**d**) Bilateral (l) [42]; (**e)** Bilateral (**g**) [42]; (**f**) $l_{0}$ minimization [47]; (**g**) Guided normal filtering [43]; (**h**) Our Approach.

In Figure 17, we present reconstructed results of different models (i.e., twelve, blocks, sharp sphere) using a variety of state-of-the-art methods. For an easier comparison among the methods, we also provide enlarge details as well as the NMSVE and mean θ metrics. Heatmap visualizations are also offered to show the distortion alleviation. The visualized results show that our approach outperforms all the other compared methods.

Table 6 presents a variety of reconstruction quality metrics for several denoising methods. By observing the quality metrics, we can verify that our method provides the best results in almost every case study. The quality of the denoising results is evaluated using a variety of different metrics that are shortly presented below:

- θ represents the angle between the normal of the ground truth face and the resulting face normals, averaged over all faces.
- Dmean d is the average distance between the vertices of the reconstructed and the original 3D mesh.
- Dmax d is the maximum among the distances of the vertices of the reconstructed and the original 3D mesh.
- dist n is the average distance between the point normals of the reconstructed and the original 3D mesh.
- Dmean n is the average distance between the face normals of the reconstructed and the original 3D mesh.
- NMSVE (Normalized Mean Square Visual Error) has been shown to correlate well with perceived distortion by measuring the average error in the Laplacian and Cartesian domains [48].

**Figure 18:**
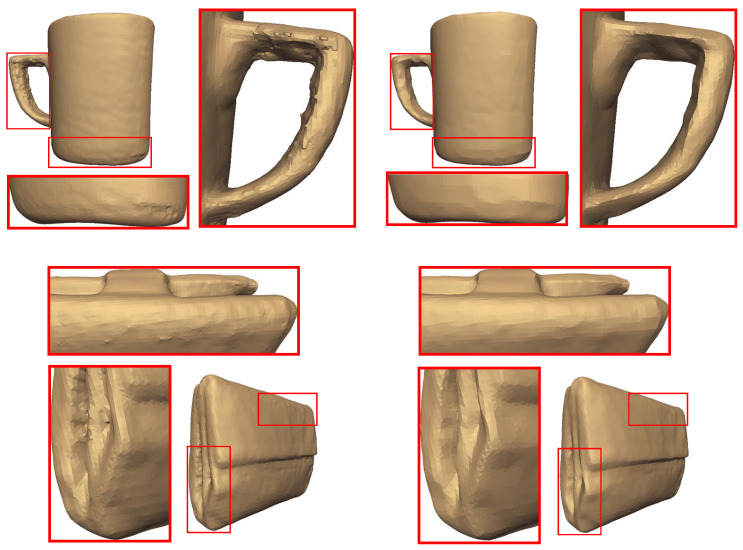
(**Left**) Three-dimensional scanned cup and wallet with abnormalities. (**Right**) Denoising results in respect of features.

We also present the denoising results of two real-scanned noisy 3D models (i.e., cup and wallet in Figure 18). Our method removes the abnormalities without over smoothing the surface of the object, preserving at the same time the high-frequency features of the objects. However, the evaluation of our method, in this case, is not feasible since the ground truth model is not known beforehand. An extended experimental analysis and results can be found in the Supplementary Materials (File S1).

## 8. Conclusions

In this work, we introduced a fast spectral processing approach ideally suited for low-level applications (i.e., denoising and compression) applied in highly dense static and dynamic 3D meshes in real-time. To overcome the high computational complexity of the SVD implementation, we exploited potential spectral coherence between different parts of a mesh and then we applied the problem of tracking graph Laplacian eigenspaces via orthogonal iterations. In the experimental analysis, we used a large variety of CAD and scanned 3D meshes. The results showed that the subspace tracking techniques allow a robust estimation of dictionaries at significantly lower execution times in comparison to the direct SVD implementations.

However, despite the better time execution performance that the orthogonal iteration approaches have, compared to the direct SVD, the careful selection of an optimal subspace size is necessary in order to simultaneously achieve both the best reconstruction quality and the fastest compression/denoising execution times. 

## Figures and Tables

**Table 1:** Mean squared error between the R[1] of different models and the mean $\tilde{R}$ of each model. The lowest value per row is highlighted in bold.

	Armadillo $\tilde{R}$	Fandisk $\tilde{R}$	Sphere $\tilde{R}$	Trim Star $\tilde{R}$	Twelve $\tilde{R}$
**Armadillo** R[1]	**0.0606**	13.9720	10.0905	1.2347	37.4199
**Fandisk** R[1]	15.6144	**1.4120**	11.1582	8.4506	29.8815
**Sphere** R[1]	10.4615	11.4700	**0.8857**	3.9065	26.4125
**Trim star** R[1]	1.3122	8.5019	3.919	**0.6095**	29.6996
**Twelve** R[1]	37.8481	30.2103	26.6648	30.0618	**4.5641**

**Table 2:** Mean Normal Difference using different number of segments (Bunny Model with 34,817 vertices). We also compare the mean normal difference by using normal average and weighted average based on the number of the connected vertices.

Number of Submeshes	Number of Vertices per Segment	MND Using Simple Average	MND Using Weighted Average
25	1392	0.0921	0.0915
40	~870	0.0931	0.0925
50	~696	0.0941	0.0934
70	~497	0.0960	0.0952
100	~348	0.0988	0.0980
200	~174	0.1039	0.1028
500	~69	0.1163	0.1150

**Table 3:** Mean normal difference using different size of equal-sized overlapped submeshes (Julio Model with 36,201 vertices 70 segments).

Type of Overlapping	Number of Vertices per Segment	Coarse Denoising MND	Fine Denoising MND
max	532	0.1248	0.1176
1.05 · max	558	0.1228	0.1173
1.10 · max	585	0.1203	0.1172
1.15 · max	611	0.1188	0.1169
1.20 · max	638	0.1174	0.1169
1.25 · max	665	0.1164	0.1164

**Table 4:** Mean normal difference using different size of equal-sized overlapped submeshes (Julio Model with 36,201 vertices 100 segments).

Type of Overlapping	Number of Vertices per Segment	Coarse Denoising MND	Fine Denoising MND
max	372	0.1276	0.1189
1.05 · max	390	0.1248	0.1187
1.10 · max	409	0.1228	0.1185
1.15 · max	427	0.1208	0.1183
1.20 · max	446	0.1184	0.1174
1.25 · max	465	0.1175	0.1168

**Table 5:** Mean normal difference using different size of equal-sized overlapped submeshes (Julio Model with 36,201 vertices 50 segments).

Type of Overlapping	Number of Vertices per Segment	Coarse Denoising MND	Fine Denoising MND
max	741	0.1229	0.1167
1.05 · max	778	0.1207	0.1166
1.10 · max	815	0.1188	0.1163
1.15 · max	852	0.1172	0.1160
1.20 · max	889	0.1160	0.1159
1.25 · max	926	0.1159	0.1158

**Table 6:** Evaluation of the experimental results using different metrics. The lowest value per each row is highlighted in bold.

	Metrics	Bilateral [41]	Non-[45] Iterative	Fast & [46] Effective	Bilateral (l) [42]	Bilateral (g) [42]	l0 min [47]	Guided Normal [43] Filtering	Our Approach
**Twelve (0.5)**	θ	11.7204	11.093	7.4519	7.3683	7.271	8.4626	2.7542	**2.668**
	Dmean d	0.017	0.0155	0.0115	0.0129	0.0123	0.0317	0.0128	**0.006**
	Dmax d	0.1357	0.1074	0.0728	0.0947	**0.0741**	0.1357	0.1594	0.0995
	dist n	0.1434	0.1301	0.1051	0.1055	0.1073	0.1518	0.0809	**0.0645**
	NMSVE	$6.98\times 10^{-5}$	$5.4\times 10^{-5}$	$4.44\times 10^{-5}$	$4.26\times 10^{-5}$	$4.84\times 10^{-5}$	$5.43\times 10^{-5}$	$5.32\times 10^{-5}$	$3.55\times 10^{-5}$
	Dmean n	0.2113	0.2002	0.1349	0.1322	0.1331	0.1627	0.0519	**0.0465**
**Block (0.4)**	θ	12.7155	13.8501	5.8023	8.0165	5.3062	4.9734	3.572	**2.9826**
	Dmean d	0.1873	0.1425	0.0857	0.096	0.0744	0.1922	0.1066	**0.0544**
	Dmax d	0.8781	0.8684	0.8206	0.7009	0.8759	0.6836	0.9967	**0.6479**
	dist n	0.236	0.2179	0.1536	0.17	0.1462	0.1911	0.1443	**0.1064**
	NMSVE	$3.26\times 10^{-5}$	$3.47\times 10^{-5}$	$2.14\times 10^{-5}$	$2.28\times 10^{-5}$	$2.1\times 10^{-5}$	$2.88\times 10^{-5}$	$2.68\times 10^{-5}$	$1.5\times 10^{-5}$
	Dmean n	0.3134	0.3106	0.1422	0.1866	0.128	0.1808	0.1131	**0.0788**
**Fandisk (0.7)**	θ	22.4862	27.9264	13.1918	15.0545	14.2553	6.2186	6.3721	**6.0669**
	Dmean d	0.0376	0.0366	0.0276	0.0321	0.0294	0.0293	0.0291	**0.0155**
	Dmax d	0.2412	0.2093	0.2048	0.188	0.1987	0.1373	0.1865	**0.1207**
	dist n	0.5803	0.6209	0.5447	0.5739	0.5739	0.4529	0.4495	**0.4065**
	NMSVE	$7.49\times 10^{-5}$	$9.68\times 10^{-5}$	$4.89\times 10^{-5}$	$4.79\times 10^{-5}$	$5.33\times 10^{-5}$	$3.5\times 10^{-5}$	$5.28\times 10^{-5}$	$3.01\times 10^{-5}$
	Dmean n	0.403	0.4937	0.2353	0.2713	0.2535	0.1221	0.119	**0.1104**

## Supplementary Materials

The following are available online at https://www.mdpi.com/2313-433X/6/6/55/s1, File S1: Supplementary material of Spectral Processing for Denoising and Compression of 3D Meshes Using Dynamic Orthogonal Iterations.
