# Supplementary material for: Spectral Processing for Denoising and Compression of 3D Meshes Using Dynamic Orthogonal Iterations
Source: J Imaging. 2020 Jun 26;6(6):55. doi: 10.3390/jimaging6060055 (PMC8321066; doi:10.3390/jimaging6060055)
Supplement: Supplementary file 1 [file jimaging-06-00055-s001.pdf]

Supplementary Material

1 Introduction

This document provides supplementary material for the original paper, entitled as "Spectral Processing for Denoising and Compression of 3D Meshes using Dynamic Orthogonal Iterations". At the following sections, we present extra experiments and discussion which are not included in the main manuscript.

2 Evaluation of different alternative steps that could be used during the coarse reconstruction approach

2.1 Different forms of weighted Laplacian matrices

In this paragraph we investigate the performance of different weighted kernels, which have been suggested by the literature, as they will be used for the creation of the weighted Laplacian matrix. More specifically, the used kernels are:

- $A = cot(\theta_{a_{ij}}) + cot(\theta_{b_{ij}})$
- $A = e^{\|\mathbf{v}_i - \mathbf{v}_j\|_2^2}$
- $A = \frac{1}{\|\mathbf{v}_i - \mathbf{v}_j\|_2^2}$

In Fig. 1, we present the reconstructed results of different models, using the aforementioned weighted Laplacian matrices. Additionally we provide heatmaps visualizing the Hausdorff distance between the reconstructed and the ground truth models. As we can see, the perceptual difference between these methods is very small and in some examples barely noticeable (especially between the cotangent approach and ours).

2.2 Different approaches to eliminate the edge effect problem

In the next Figs. 2-3, we present the coarse reconstructed results of different reconstruction approaches. We assume that the used model (fandisk) has been affected by different levels of Gaussian noise (i.e., $\sigma_E = 0.1$, $\sigma_E = 0.2$). Also, it has been used in different segmentation scenarios. As we can see, observing the results, the edge effect is apparent in areas where submeshes are connected, especially for the non overlapping case. In the simple overlapping case, the edge effect has been mitigated but has not been eliminated yet. In the weighted overlapping case, the results seem to be independent and unaffected of the partitioning. Finally, in the overlapping case, taking into account the highest value, the results are similar or worse of the simple overlapping case.

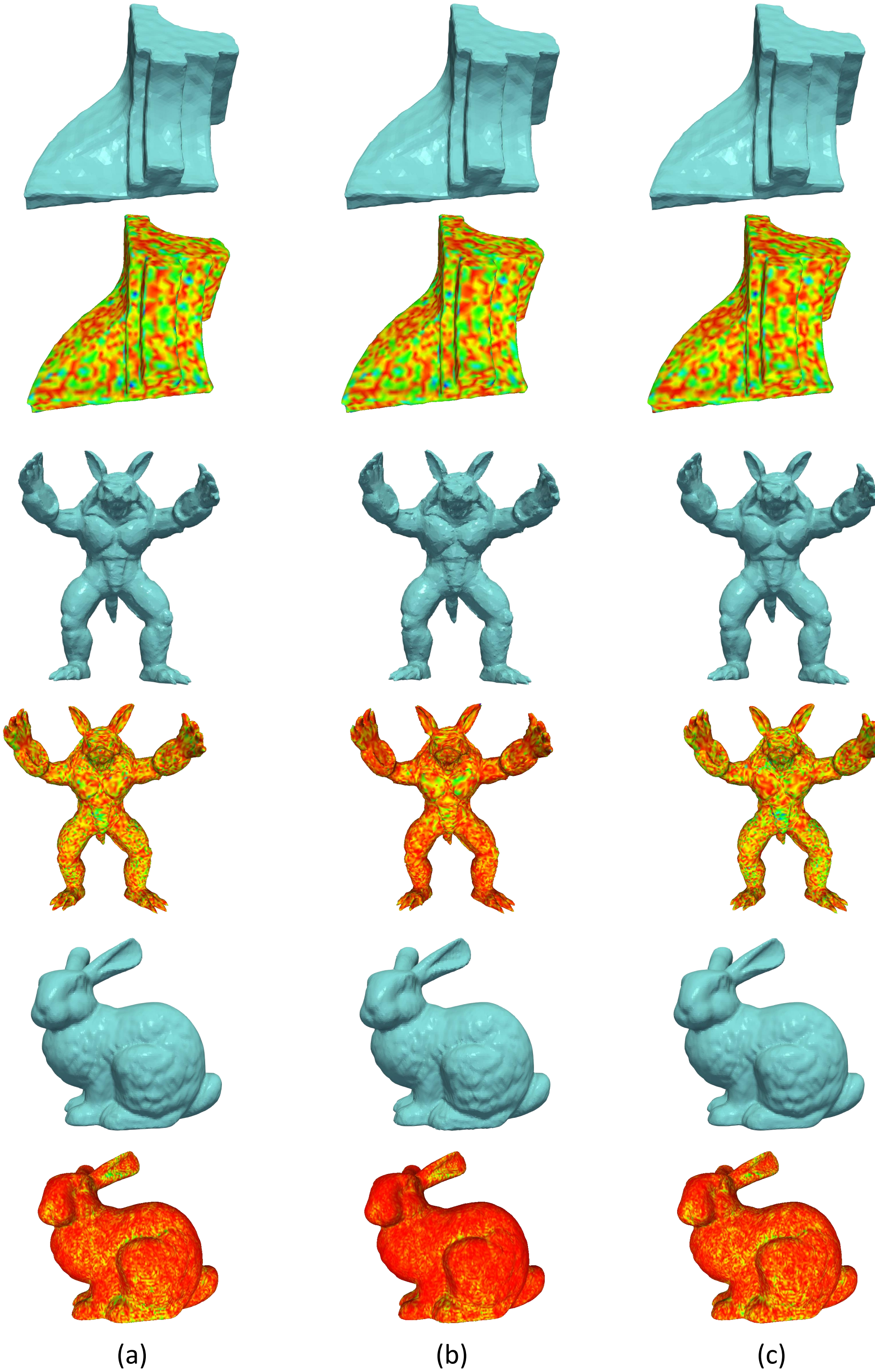

Figure 1: Reconstructed results and heatmaps of Hausdorff distance for different weighted Laplacian matrices (a) cotangent weight, (b) exponential weights, (c) our approach.

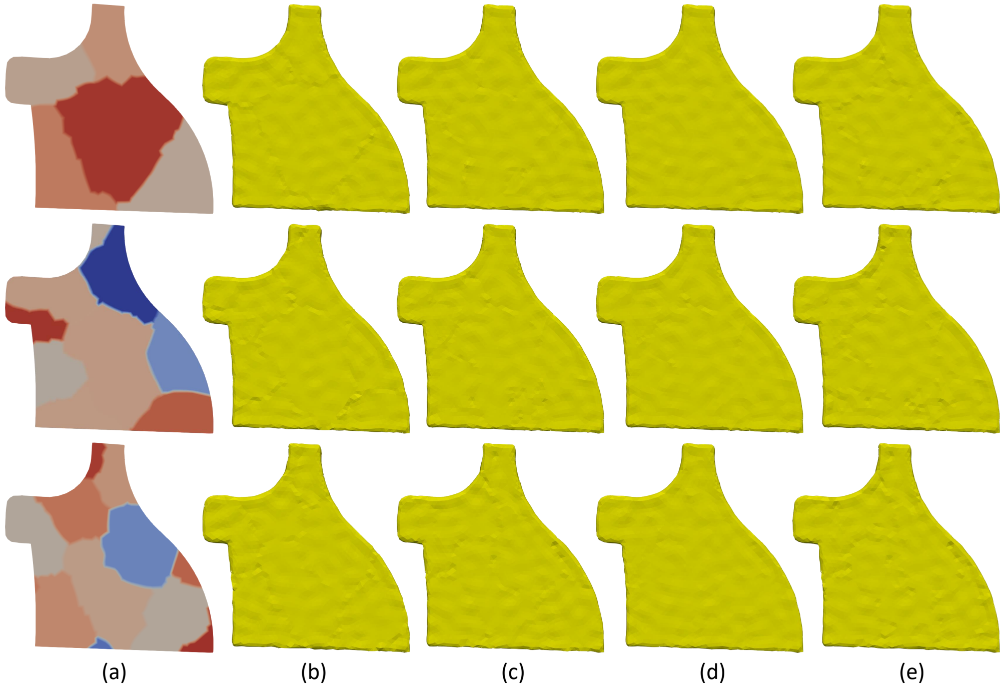

Figure 2: Coarse reconstructed results of Fandisk model affected by Gaussian noise $\sigma_E = 0.1$. (a) Metis segmentation in 10, 15 and 20 submeshes respectively, (b) non overlapping case, (c) overlapping case, (d) weighted overlapping case (e) overlapping case taking the highest value.

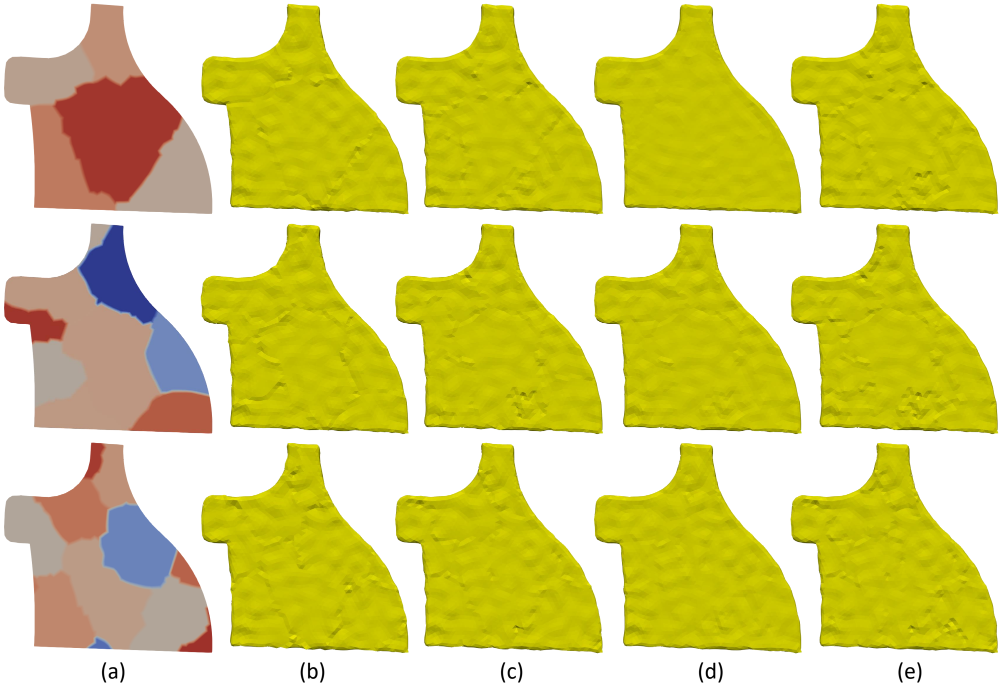

Figure 3: Coarse reconstructed results of Fandisk model affected by Gaussian noise $\sigma_E = 0.2$. (a) Metis segmentation in 10, 15 and 20 submeshes respectively, (b) non overlapping case, (c) overlapping case, (d) weighted overlapping case (e) overlapping case taking the highest value.

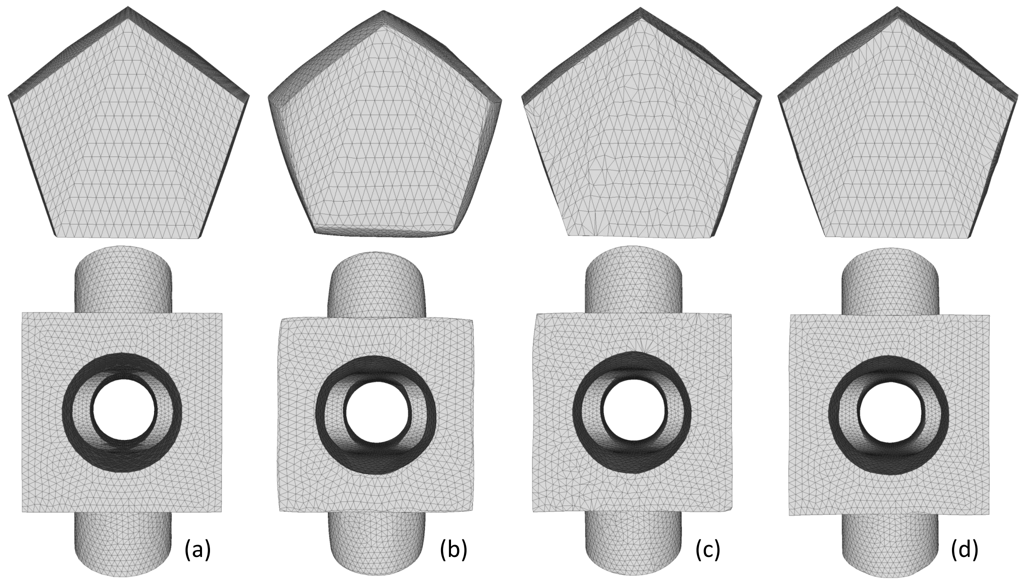

Figure 4: (a) Original mesh, (b) l_0 minimization [2], (c) guided normals filtering [5], (d) our approach.

3 Experimental Results

One significant advantage of our method, in contrary to the other methods, is the fact that it does not negatively affect the relative position of vertices. Fig. 4 shows the reconstructed results, emphasizing at the triangulated faces for different denoising methods. It can be easily observed that other state-of-the-art approaches significantly modify the relative position of the vertices, even in cases where the reconstructed quality is considered acceptable. On the contrary, our approach provides both accurate reconstruction preserving the real distance between adjacent nodes.

In Fig. 5, we present the reconstructed results of noisy models (i.e., block and fandisk with Gaussian noise $\mathcal{N}(0, 0.7)$) for different state-of-the-art methods with (second row) and without (first row) using our proposed method as a preprocessing step. The results verify our assumption that the proposed OI approach can optimize both the reconstruction quality and the computational complexity of these methods since the use of the coarse step significantly accelerates the convergence of the fine denoising step, reducing the vertex update iterations required for achieving a specific reconstruction quality.

The experiments have shown that the reconstructed results are affected by the number of iterations or/and the power z of matrix $\mathbf{R}$. In Fig. 6, we present the coarse denoised results of "Twelve" model using OI with different values of z. Higher values of z are related to higher accuracy. As we can see, the results seem to be identical with this of the direct SVD for OI with $z > 4$. In Table 1, we present the reconstruction quality, evaluated by the metric θ, and the execution times (lower value of z results in faster executions) between the direct SVD implementation and different approaches of OI using different power z of matrix $\mathbf{R}$.

Our method provides high quality reconstruction results even in cases where the noise and features are extremely difficult to be separated, as presented in Fig. 7.

Fig. 8 presents the NMSVE metric for the reconstructed models while the remaining number of eigenvalues changes. Similar conclusions with the compression use case, we can draw in this denoising application too. More specifically, it seems that the OI can achieve almost the same reconstructed quality with the direct SVD method but very faster (almost 24 times).

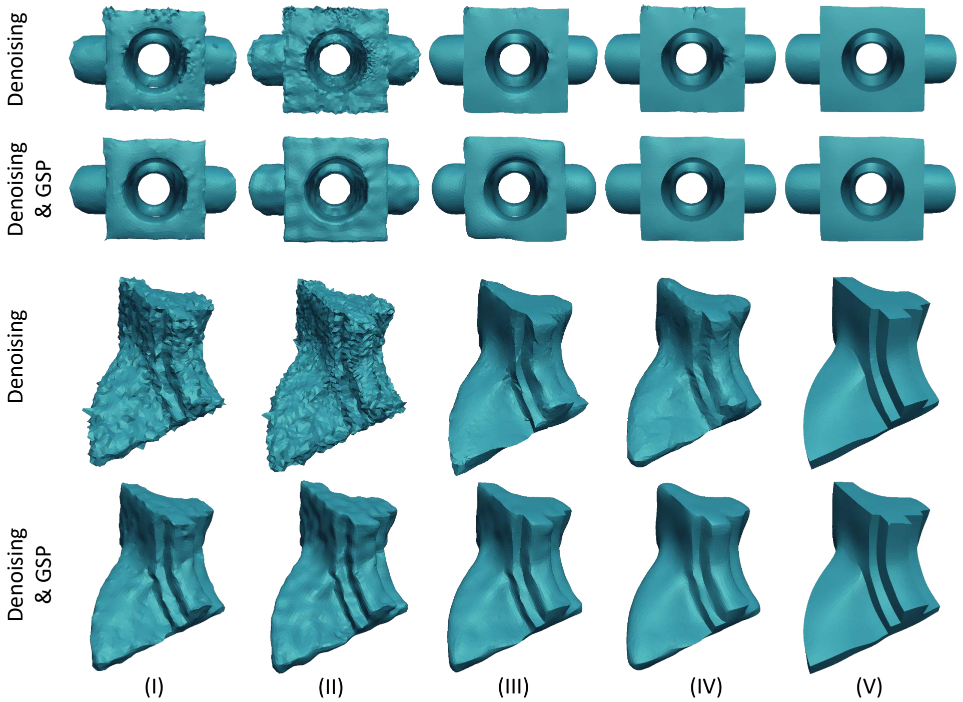

Figure 5: [First lines] Original reconstructed denoising results and [Second lines] reconstructed denoising results after applying the proposed Graph Spectral Processing (GSP) as a pre-processing step, for different methods: (i) bilateral mesh denoising[1], (ii) non-iterative feature preserving smoothing [3], (iii) fast and effective feature preserving denoising [4], (iv) bilateral normal filtering [6], (v) guided normal filtering [5].

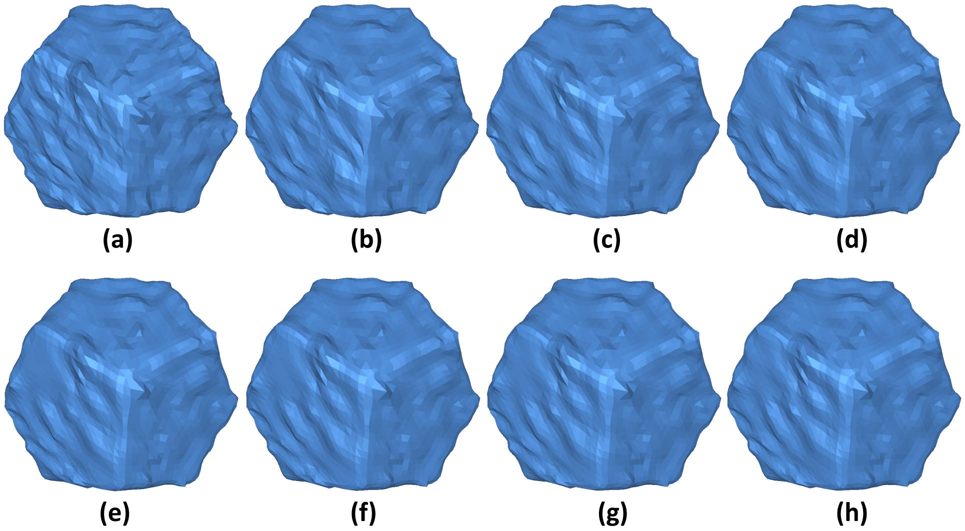

Figure 6: Reconstructed models, after the coarse denoising step, using different types of $\mathbf{R}^z$, (a) $z = 1$, (b) $z = 2$, (c) $z = 3$, (d) $z = 4$, (e) $z = 5$, (f) $z = 6$, (g) $z = 7$, (h) SVD.

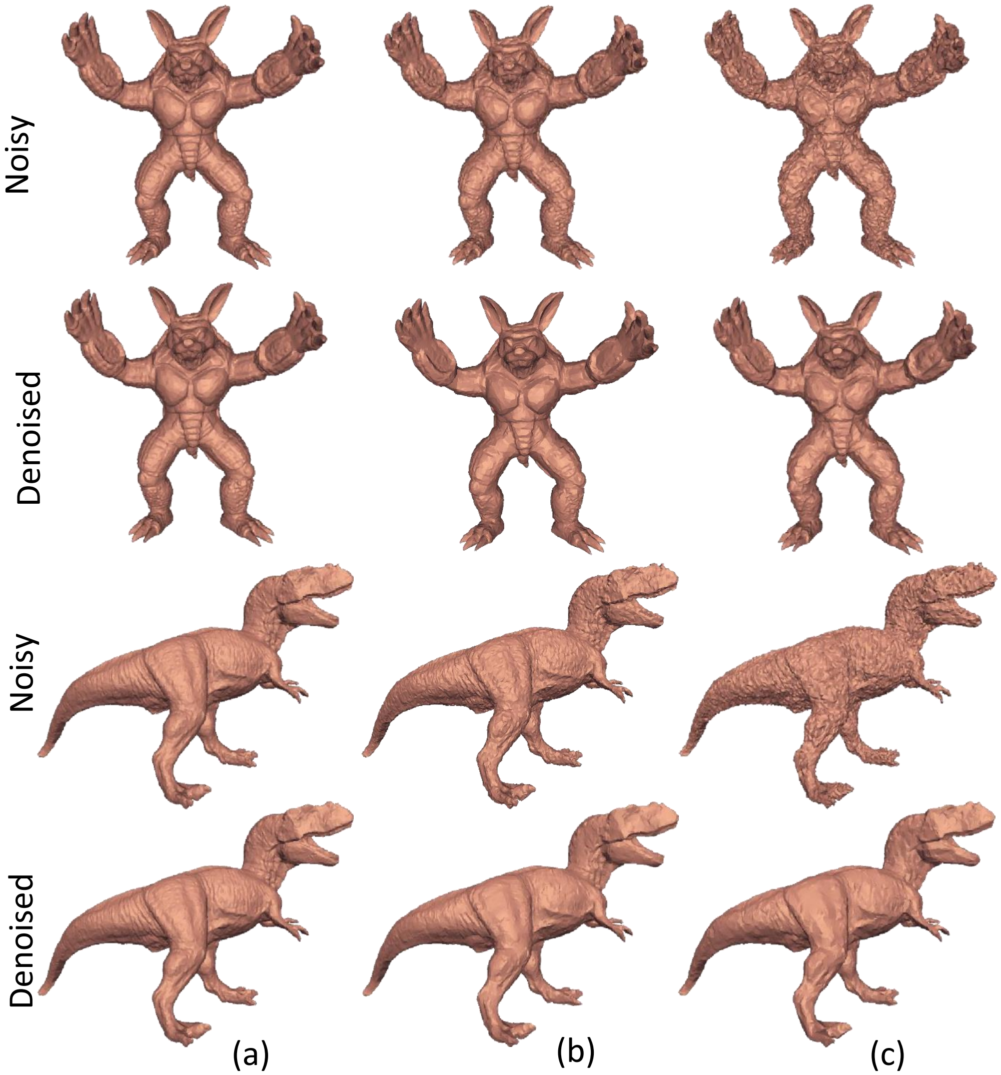

Figure 7: Denoising results for a variety of noise patterns (a) $\sigma_E = 0.05$, (b) $\sigma_E = 0.10$, (c) $\sigma_E = 0.15$ in 3D models (armadillo and tyra) consisting of many and different-scaled geometric features.

	**Twelve**		**Fandisk**	
	t	θ	t	θ
$\mathbf{R}^1$	0.031	11.57	0.077	16.36
$\mathbf{R}^2$	0.049	10.26	0.110	14.75
$\mathbf{R}^3$	0.099	13.97	0.170	14.54
$\mathbf{R}^4$	0.114	13.84	0.202	14.54
$\mathbf{R}^5$	0.136	13.7	0.242	14.44
$\mathbf{R}^6$	0.142	13.59	0.297	14.5
$\mathbf{R}^7$	0.157	13.57	0.313	14.52
$\mathbf{R}^8$	0.184	13.55	0.355	14.84
$\mathbf{R}^9$	0.201	13.53	0.407	15.6
SVD	0.901	9.83	1.953	14.56

Table 1: Execution times and corresponding values of the metric θ using different types of $\mathbf{R}^z$ and SVD.

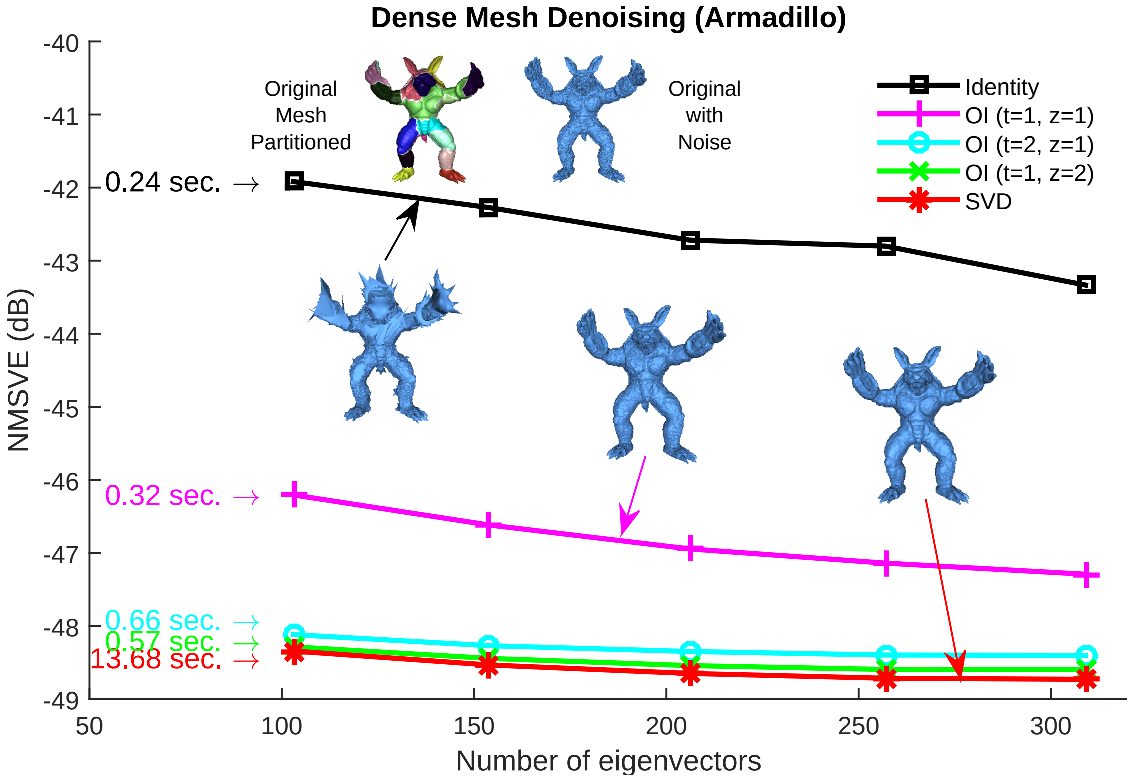

Figure 8: NMSVE of the reconstructed models per different remaining number of eigenvalues for different compared approaches.

References

[1] Shachar Fleishman, Iddo Drori, and Daniel Cohen-Or. Bilateral mesh denoising. *ACM Trans. Graph.*, 22(3):950–953, jul 2003.

[2] Lei He and Scott Schaefer. Mesh denoising via l0 minimization. *ACM Trans. Graph*, 32(4):64:1–64:8, 2013.

[3] Thouis R. Jones, Fredo Durand, and Mathieu Desbrum. Non-iterative, feature-preserving mesh smoothing. *ACM Trans. Graph*, 22(3):943–949, 2003.

[4] Xianfang Sun, Paul L. Rosin, Ralph R. Martin, and Frank C. Langbein. Fast and effective feature-preserving mesh denoising. *IEEE TRANSACTIONS ON VISUALIZATION AND COMPUTER GRAPHICS*, 13(5):925–938, 2007.

[5] Wangyu Zhang, Bailin Deng, Juyong Zhang, Sofien Bouaziz, and Ligang Liu. Guided mesh normal filtering. *Pacific Graphics*, 34(7), 2015.

[6] Youyi Zheng, Hongbo Fu, Oscar Kin-Chung Au, and Chiew-Lan Tai. Bilateral normal filtering for mesh denoising. *IEEE Trans. Vis. Comput. Graph.*, 17(10):1521–1530, 2011.
